# Association Between Pre‐Diagnostic Delay and Survival Among Patients With Esophageal and Gastric Cancer Treated With Curative Intent During the COVID19 Pandemic

**DOI:** 10.1002/cam4.70939

**Published:** 2025-05-09

**Authors:** Xin Wang, Yvonne Bach, Katherine Lajkosz, Osvaldo Espin‐Garcia, Hiroko Aoyama, Michael Wang, Ronan McLaughlin, Lucy Ma, Carly Barron, Farooq Abdul Rehman, Eric Xueyu Chen, Johnathan Chi‐Wai Yeung, Carol J. Swallow, Savtaj Brar, Rebecca Wong, Aruz Mesci, John Kim, Patrick Veit‐Haibach, Sangeetha Kalimuthu, Raymond Woo‐Jun Jang, Elena Elimova

**Affiliations:** ^1^ Division of Medical Oncology Princess Margaret Cancer Centre, University Health Network Toronto Ontario Canada; ^2^ Eliot Phillipson Clinician‐Scientist Training Program University of Toronto Toronto Ontario Canada; ^3^ Department of Biostatistics Princess Margaret Cancer Centre, University Health Network Toronto Ontario Canada; ^4^ Epidemiology and Biostatistics Schulich School of Medicine and Dentistry London Ontario Canada; ^5^ Department of Bio‐Medical Science Guelph University Guelph Ontario Canada; ^6^ Division of Thoracic Surgery Toronto General Hospital, University Health Network Toronto Ontario Canada; ^7^ Department of Surgical Oncology Princess Margaret Cancer Centre, University Health Network Toronto Ontario Canada; ^8^ Department of Surgery Mount Sinai Hospital Toronto Ontario Canada; ^9^ Division of Radiation Oncology Princess Margaret Hospital, University Health Network Toronto Ontario Canada; ^10^ Joint Department of Medical Imaging Toronto General Hospital, University Health Network Toronto Ontario Canada; ^11^ Division of Pathology Toronto General Hospital, University Health Network Toronto Ontario Canada

**Keywords:** COVID19, diagnosis, gastric cancer, gastroesophageal cancer

## Abstract

**Background:**

The majority of esophageal and gastric cancers are diagnosed at an advanced stage with poor overall survival (OS). Whether the pre‐diagnostic interval from symptom onset has any impact on OS is unclear. We investigated this question in the peri‐COVID19 pandemic era.

**Methods:**

We retrospectively analyzed a cohort of 308 patients with esophageal, gastroesophageal junction, or gastric carcinoma treated with curative intent at the Princess Margaret Cancer Centre from January 2017 to December 2021. Clinical details pertaining to the initial presentation were determined through a retrospective chart review. Cox proportional hazards regression models were used to assess the association between pre‐diagnostic intervals and OS, adjusting for baseline patient characteristics.

**Results:**

The median interval from symptom onset to diagnosis was 98 days (IQR 47–169 days). Using a cox proportional hazard model, prolonged pre‐diagnostic interval was not associated with worse OS (HR 1.00, *p* = 0.62). Comparing patients diagnosed before and during the COVID19 pandemic, there was a notable increase in diagnostic delay with median pre‐diagnostic interval increasing from 92 to 126 days (*p* = 0.007). Median age at time of diagnosis was 69.6 during the pandemic vs. 64.7 before the pandemic. Linear regression showed squamous cell histology was significantly associated with increasing time to initial diagnosis (*p* = 0.04), but this did not hold true in a multivariable model. Looking at other delay metrics, there were no changes in time interval from diagnosis to treatment during versus before the pandemic (median = 1.7 weeks for both), and there was no change in time from diagnosis to resection in those patients who underwent surgery.

**Conclusion:**

The COVID19 pandemic caused significant diagnostic delay for patients presenting with curative gastroesophageal and gastric cancer. The lack of correlation of pre‐diagnostic interval with OS may reflect underlying tumor biology as the driving force that determines prognosis.

## Graphical abstract

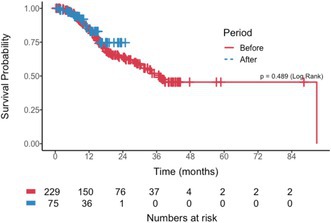

## 1 Introduction

The Coronavirus Disease 2019 (COVID‐19) global pandemic has and continues to greatly impact cancer care delivery around the world [1]. Since its declaration on March 11, 2020, by the World Health Organization, drastic measures have been introduced to contain the virus's spread. Protocols were adapted to offset the risk of surgical delay, especially for curative intent patients [2]. Population‐based cohort studies have shown that more than 20% of cancer care services were delayed or canceled, leading to unprecedented backlogs in cancer care [3]. Around the globe, cancer screening was severely impacted, and the true scope of this impact remains unknown [4, 5].

Gastroesophageal cancers remain a significant global burden and portend an overall poor prognosis, with median overall survival for advanced disease around one year [6, 7]. Significant effort has therefore been invested in cancer screening to detect early stage disease and reduce the delay to diagnosis [8, 9, 10]. In a meta‐analysis of more than 1 million patients that did not include gastroesophageal cancer, delay in treatment is associated with increased mortality [11]. Among patients with esophageal cancer, traditional mantra stipulates that a delay in diagnosis may have an impact on upstaging their cancer, presumably resulting in worse prognosis [9]. More recent cohorts reported conflicting evidence; in a retrospective study of 3613 patients, time to diagnosis defined as days from first symptom onset to diagnosis, did not affect resectability, postoperative morbidity, or survival [10]. Another large retrospective single‐center experience of 671 gastric cancer patients showed that neither patient delay, defined as symptom onset to first health care provider encounter, nor diagnostic delay had any impact on overall survival [12].

The COVID‐19 pandemic presented a microcosm to study the impact of pre‐diagnostic interval on clinical outcomes for patients with esophageal and gastric cancer. Around the world, COVID‐19 has led to a significant decline in endoscopic procedures and other screening tests [3, 13]; the clinical impact of this among gastroesophageal patients is lacking in the literature, especially in a publicly funded health system [14, 15]. Studies from National Health Services in Scotland have recently reported increased time to gastroscopy, worse performance status, and a higher stage of disease after COVID‐19 lockdown contributing to a worse overall survival [16]. This study did not exclusively examine patients in the curative setting. In another study from the Netherlands, the number of esophagogastric cancer diagnoses declined during the COVID‐19 pandemic, while an increased percentage of patients was diagnosed with incurable disease [17]. Given the conflicting evidence, we designed this retrospective cohort study to determine whether the COVID‐19 pandemic caused any increase in pre‐diagnostic interval among patients with esophageal, gastroesophageal junction, and gastric cancers treated with curative intent and if this contributed to worse survival outcomes.

## 2 Methods

### 2.1 Study Design and Population

This was a retrospective cohort study of all consecutive adult patients (≥ 18 years old) who were treated for their gastroesophageal (GE) cancer at the Princess Margaret Cancer Centre (PMCC), Toronto, Canada, from January 2017 to December 2021. Patients were identified from an institutional registry approved by the University Health Network Research Ethics Board (20‐5443) and were included if they: (1) had histologically proven localized/locally advanced gastric, gastroesophageal junction (GEJ), or esophageal squamous cell carcinoma or adenocarcinoma, and (2) were initially treated with curative intent therapy. An informed consent waiver was granted by the research ethics board for this study.

Patients were stratified into two cohorts based on their date of presentation to the PMCC: (1) patients who presented before March 11, 2020 (date of pandemic declaration by WHO), and (2) patients seen during the pandemic (i.e., presented on or after March 11, 2020). The pre‐diagnostic interval was defined as the time period between patient‐reported symptom onset and pathologic diagnosis. Symptoms ranged from dysphagia, weight loss, abdominal pain, nausea/vomiting, melena, hematemesis, or early satiety. We used the middle of the month/season for records where only a non‐specific date was documented.

### 2.2 Data Collection

Patient demographics, clinicopathologic characteristics, treatment details, and follow‐up data were abstracted from the institution's electronic medical records system and entered into REDCap (Research Electronic Data Capture). The accuracy of data abstraction was independently verified by two research coordinators.

### 2.3 Outcomes

The primary outcome was overall survival (OS), defined as the time from pathologic diagnosis to death from any cause. The last date of follow‐up was March 22, 2022. Patients without documented evidence of an event were censored at the date of last follow‐up.

### 2.4 Statistical Analysis

Pre‐diagnostic interval and cohort characteristics were summarized using descriptive statistics and stratified by time period. Differences in the distribution of continuous and categorical characteristics by time period were assessed using Mann–Whitney *U* and Chi‐squared tests, respectively.

The Kaplan–Meier method was used to summarize OS by group, and the log‐rank test was used to compare differences in OS by time period. Univariable and multivariable Cox proportional hazards regression models were used to assess the association between pre‐diagnostic interval (modeled as a continuous predictor), patient characteristics, and OS. The model was adjusted for the following variables determined a priori based on existing literature: age, sex, body mass index (BMI), alcohol consumption, smoking history, Eastern Cooperative Oncology Group (ECOG) performance status, clinical stage, histology, and tumor grade. To assess the proportional hazards assumptions, the Schoenfeld residuals were plotted and examined for each predictor. Univariable and multivariable linear regression models, using the pre‐diagnostic interval as outcome and the aforementioned covariates of interest as predictors, were used to assess factors contributing to an increase in pre‐diagnostic interval. Because the pre‐diagnostic interval was skewed in distribution, it was log‐transformed for the regression models. A two‐sided statistical significance level of 5% (*p* < 0.05) was used. Statistical analyses were performed using R v4.1.2. Complete case analyses were performed, and missing data were not imputed.

## 3 Results

### 3.1 Study Population

From January 2017 to December 2021, 308 gastroesophageal cancer patients were treated with curative intent modalities at PMCC. Baseline demographics are shown in Table 1. The median age of the cohort at presentation was 66 years (interquartile range; IQR = 59–73 years) and 70% (*n* = 217) were male. The median interval from symptom onset to diagnosis was 98 days (IQR = 47–169 days). The cohort was stratified into two subgroups: (1) Pre‐COVID subgroup (*n* = 229; 74%) defined as patients seen before the start of the COVID‐19 pandemic (March 11, 2020), and (2) COVID subgroup (*n* = 79; 26%) for those seen during the pandemic (on or after March 11, 2020).

**TABLE 1:** Baseline characteristics.

	Cohort (*n* = 308)	Pre‐COVID (*n* = 229)	COVID (*n* = 79)	*p*
Pre‐diagnostic interval, days				
Mean (SD)	150.8 (199.6)	149.3 (220.7)	154.9 (119.8)	**0.007**
Median (IQR)	98 (47–169)	92 (42–153)	126.0 (65–227)	
Age at presentation, years				
Mean (SD)	65.8 (11.0)	64.6 (11.0)	69.3 (10.2)	**0.002**
Median (IQR)	66.4 (59.0–73.4)	64.7 (58.0–72.6)	69.6 (63.3–76.1)	
Sex, male	217 (70)	159 (69)	58 (73)	0.60
Race, Asian	48 (16)	33 (14)	15 (19)	0.43
BMI	*n* = 298	*n* = 215	*n* = 74	0.25
Median (IQR)	24.5 (21.9–28.0)	24.9 (21.9–28.3)	23.8 (22.5–26.8)	
ECOG, *n* (%)	*n* = 300	*n* = 221	*n* = 79	0.08
0	75 (25)	59 (27)	17 (20)	
1	195 (65)	143 (65)	52 (66)	
2	22 (7)	16 (7)	6 (8)	
3	7 (2)	2 (1)	5 (6)	
4	1 (0)	1 (0)	0 (0)	
Primary tumor location, *n* (%)				
Esophagus	84 (27)	52 (23)	32 (41)	**0.003**
AEG1	71 (23)	50 (22)	21 (27)	
AEG2	42 (14)	36 (16)	6 (8)	
AEG3	22 (7)	15 (7)	7 (9)	
Gastric	89 (29)	76 (33)	13 (16)	
Nodal status, *n* (%)				
N0	107 (35)	80 (35)	27 (34)	0.78
N+	172 (56)	129 (56)	43 (54)	
Unknown	29 (9)	20 (9)	9 (11)	
Stage, *n* (%)				
0	18 (6)	16 (7)	2 (3)	0.48
I	39 (13)	26 (11)	13 (16)	
II	72 (23)	56 (24)	16 (20)	
III	128 (42)	94 (41)	34 (43)	
IV	12 (4)	10 (4)	2 (3)	
Unknown	39 (13)	27 (12)	12 (15)	
Tumor histology, *n* (%)				
Adenocarcinoma	235 (76)	181 (79)	54 (68)	0.08
Squamous cell carcinoma	73 (24)	48 (21)	25 (32)	
Tumor grade, *n* (%)	*n* = 307	*n* = 228	*n* = 79	**0.04**
G1	17 (6)	16 (7)	1 (1)	
G2	106 (35)	76 (33)	30 (38)	
G3	119 (39)	94 (41)	25 (32)	
GX	65 (21)	42 (18)	(29)	
Alcohol, *n* (%)				
Frequent	58 (19)	40 (17)	18 (23)	0.09
Never	105 (34)	75 (33)	30 (38)	
Occasional	81 (26)	58 (25)	23 (29)	
Past	48 (16)	41 (18)	7 (9)	
Unknown	16 (5)	15 (7)	1 (1)	
Smoking, *n* (%)				
Current smoker	45 (15)	33 (14)	12 (15)	0.47
Ex‐smoker	133 (43)	96 (42)	37 (47)	
Never smoker	123 (40)	93 (41)	30 (38)	
Unknown	7 (2)	7 (3)	0 (0)	

*Note:* Bold values represent significant *p* values where *p* is < 0.05.

Abbreviations: BMI = body mass index, ECOG = Eastern Cooperative Oncology Group.

### 3.2 Patients Seen Before and During the COVID‐19 Pandemic

Compared to patients seen before the pandemic, the pre‐diagnostic interval from symptom onset to pathologic diagnosis was significantly increased for those who presented during the pandemic (median delay = 92 vs. 126 days, respectively) (*p* = 0.007, Table 1). The COVID subgroup was also significantly older (median age = 70 vs. 65 years, *p* = 0.002) and had predominantly a GEJ/esophageal primary rather than gastric (85% vs. 68%, *p* = 0.003). Tumor grading was also statistically different between the two subgroups (*p* = 0.04). Other baseline clinical characteristics were similar between these two time periods.

### 3.3 Overall Survival

**FIGURE 1:**
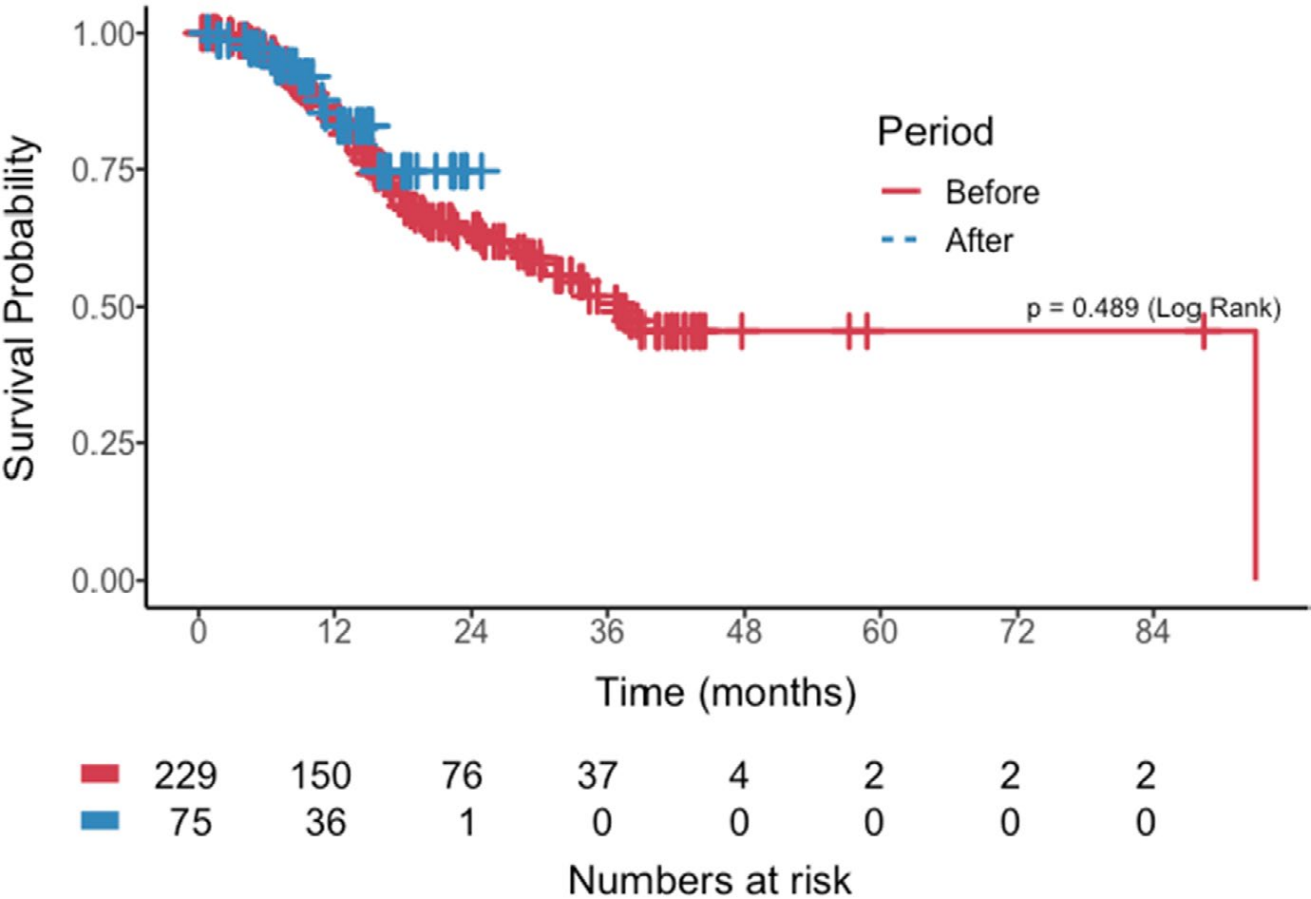
Kaplan–Meier overall survival curve among patients diagnosed pre‐ and during the COVID‐19 pandemic (*n* = 304).

The median overall survival for the cohort was 37.8 months (95% CI: 30.6‐NA months). There was no statistically significant difference in overall survival between patients seen before and during the COVID‐19 pandemic (log‐rank *p* = 0.49), as shown in Figure 1. We explored this further using both univariable and multivariable Cox proportional hazards models. Pre‐diagnostic interval as a continuous covariate was not associated with worse OS (adjusted HR = 1.00; 95% confidence interval (CI) = 1.00–1.00; *p* = 0.16) (Table 2). However, known prognostic factors such as increased age (adjusted HR = 1.04; 95% CI =1.01–1.06; *p* = 0.006), esophageal location (*p* = 0.020), and positive nodal status (adjusted HR = 3.00; 95% CI = 1.25–7.21; *p* = 0.01) were independently associated with worse OS in the multivariable model. The proportional hazards assumption, as determined by Schoenfeld residuals, holds for all variables incorporated into the model (*p* > 0.05). We next attempted to determine clinical factors that may have contributed to an increase in the pre‐diagnostic interval. A multivariable linear regression model revealed no statistically significant factors associated with increasing pre‐diagnostic interval (Table 3).

**TABLE 2:** Uni‐ and multivariable cox proportional hazards model for overall survival.

Covariable	Univariable	*p*	Multivariable	*p*
	HR (95% CI)		Adjusted HR (95% CI)	
Pre‐diagnostic interval, days	1.00 (1.00–1.00)	0.62	1.00 (1.00–1.00)	0.16
Age at presentation	1.02 (1.00–1.04)	0.052	**1.04 (1.01–1.06)**	**0.006**
Sex, male	1.06 (0.67–1.67)	0.81	1.23 (0.73–2.07)	0.43
Race, Non‐Asian	2.06 (0.95–4.46)	0.07	1.46 (0.65–3.30)	0.36
BMI	0.98 (0.95–1.03)	0.47	1.01 (0.96–1.06)	0.67
ECOG, 2–4	1.91 (0.98–3.69)	0.055	1.35 (0.60–2.99)	0.47
Siewart type (GEJ as reference)		**0.004**		**0.028**
Esophagus	1.16 (0.72–1.87)	0.53	1.41 (0.62–3.20)	0.41
Gastric	0.47 (0.26–0.82)	**0.009**	0.44 (0.23–0.85)	**0.021**
Stage group (0‐II as reference)		**< 0.001**		0.12
III‐IV	2.52 (1.54–4.13)	**< 0.001**	1.62 (0.71–3.67)	0.25
Unknown	3.70 (1.79–7.66)	**< 0.001**	3.05 (1.09–8.55)	**0.031**
Nodal status (N0 as reference)		**< 0.001**		**0.020**
N+	3.35 (1.91–5.87)	**< 0.001**	3.00 (1.25–7.21)	**0.011**
NX	2.43 (0.94–6.25)	0.067	1.30 (0.35–4.90)	0.70
Tumor histology, SCC (AC as reference)	**1.65 (1.06–2.57)**	**0.027**	1.47 (0.59–3.66)	0.41
Tumor grade (G1 as reference)				0.90
G2	0.71 (0.27–1.85)	0.48	0.68 (0.24–1.92)	0.47
G3	0.79 (0.31–2.04)	0.63	0.76 (0.27–2.16)	0.61
GX	1.02 (0.39–2.72)	0.96	0.76 (0.27–2.14)	0.60
Surgery, yes	0.24 (0.15–0.38)	**< 0.001**	—	—
Chemotherapy, yes	**0.58 (0.36–0.94)**	**0.026**	—	—
Radiation, yes	0.83 (0.54–1.28)	0.40	—	—

*Note:* Bold values represent significant *p* values where *p* is < 0.05.

Abbreviations: AC = adenocarcinoma, BMI = body mass index, ECOG = Eastern Cooperative Oncology Group, SCC = squamous cell carcinoma.

**TABLE 3:** Uni‐ and multivariable analyses for factors contributing to increasing pre‐diagnostic interval.

Covariable	Univariable	*p*	Multivariable	*p*	*n*
	Estimate (95% CI)		Estimate (95% CI)		
Age at presentation	0.00–4.8e‐03 (−0.02, 7.3e‐030.00)	0.44	0.01–7.7e‐03 (−0.02, 0.006.2e‐03)	0.27	304
Sex, male	−0.15 (−0.44, 0.14)	0.31	−0.20 (−0.53, 0.12)	0.22	304
Race, Non‐Asian	−0.05 (−0.41, 0.32)	0.80	−0.23 (−0.65, 0.19)	0.28	304
BMI	−0.01 (−0.04, 0.01)	0.33	−0.0021.8e‐03 (−0.03, 0.03)	0.91	285
ECOG, 2–4	0.02 (−0.43, 0.47)	0.93	0.04 (−0.46, 0.54)	0.87	296
Siewart class type (GEJ as reference)		0.070		0.19	304
Esophagus	0.11 (−0.21, 0.43)	0.49	−0.17 (−0.67, 0.33)	0.50	83
Gastric	−0.29 (−0.60, 0.03)	0.08	−0.33 (−0.69, 0.03)	0.070	86
Stage group (0‐II as reference)		0.26		0.38	304
II‐IV	0.13 (−0.15, 0.42)	0.36	0.28 (−0.19, 0.75)	0.24	139
Unknown	−0.21 (−0.63, 0.22)	0.33	−0.02 (−0.60, 0.56)	0.95	39
Nodal status (N0 as reference)		0.31		0.72	304
N+	−0.04 (−0.32, 0.25)	0.80	−0.10 (−0.57, 0.37)	0.68	171
NX	−0.37 (−0.86, 0.11)	0.13	−0.28 (−0.97, 0.41)	0.42	28
Tumor histology, SCC (AC as reference)	0.32 (0.01, 0.63)	0.040	0.31 (−0.21, 0.84)	0.24	304
Tumor grade (G1 as reference)		0.55		0.78	303
G2	0.16 (−0.44, 0.76)	0.60	−0.01‐9.3e‐03 (−0.69, 0.67)	0.98	105
G3	−0.02 (−0.62, 0.58)	0.95	−0.18 (−0.86, 0.51)	0.61	117
GX	0.20 (−0.42, 0.83)	0.52	−0.03 (−0.74, 0.67)	0.92	64
Surgery, yes	0.18 (−0.16, 0.51)	0.29	—	—	
Chemotherapy, yes	0.04 (−0.26, 0.35)	0.78	—	—	
Radiation, yes	0.14 (−0.13, 0.41)	0.30	—	—	

Abbreviations: AC = adenocarcinoma, BMI = body mass index, ECOG = Eastern Cooperative Oncology Group, SCC = squamous cell carcinoma.

### 3.4 Other Metrics of Health‐System Delays

The time between pathologic diagnosis and treatment initiation was also measured and compared between the pre‐COVID and COVID subgroups (Table 4). There were no significant differences in time from diagnosis to receiving treatment of any kind (median time interval = 1.7 weeks for both subgroups, *p* = 0.58) nor time from diagnosis to surgical resection (median time interval = 4.2 vs. 4.1 weeks, *p* = 0.54). Compared to esophageal and GEJ cancer patients seen prior to the start of COVID‐19, the time from diagnosis to completing a PET/CT scan was similar to patients seen during the pandemic (median time interval = 0.7 vs. 0.8 weeks, *p* = 0.21).

**TABLE 4:** Performance metric of other health‐system delays (time from diagnosis to any treatment, operation, and PET/CT scan).

	Cohort	*n*	Pre‐COVID	*n*	COVID	*n*	*p*
Time to any treatment, median (IQR), weeks	1.7 (1.2–2.3)	283	1.7 (1.2–2.4)	207	1.7 (1.3–2.0)	76	0.58
Time to surgical resection, median (IQR), weeks	4.1 (2.5–5.0)	231	4.2 (2.3–5.2)	178	5.1 (3.2–4.6)	53	0.54
Time to PET/CT^a^, median (IQR), weeks	0.7 (0.4–1.0)	195	0.7 (0.5–1.0)	153	0.6 (0.3–1.0)	66	0.21

Abbreviation: IQR = interquartile range.

^a^ Applicable to only esophageal/GEJ cancer patients who require staging PET/CT as standard‐of‐care (*n* = 219, 24 missing values).

## 4 Discussion

Profound changes to cancer care delivery occurred during the COVID‐19 pandemic, spanning from screening to supportive care [3]. The impact of these drastic shifts in models of care needs to be studied. Resectable gastroesophageal cancer requires multidisciplinary collaboration and represents a good case study of the impact of COVID‐19‐related disruptions to cancer care delivery. This study demonstrated a significant increase in pre‐diagnostic interval at a large regional cancer centre (from a median of 92 to 126 days) during the COVID‐19 pandemic compared to baseline. This delay may have stemmed from a lack of available health care due to resource reallocation, as well as fear of contracting COVID‐19, resulting in reluctance to seek medical attention [18]. Surprisingly, despite an increase in pre‐diagnostic interval, in our multivariable cox proportional hazards model, pre‐diagnostic interval was not associated with worse overall survival (HR 1.00, *p* = 0.16); however, ongoing follow‐up is necessary before any conclusions can be drawn. Our model included known prognostic factors such as age, histology, nodal status, and stage, which further supported our finding that the pre‐diagnostic interval was not associated with overall survival. Using a linear regression model, we were not able to find any known clinical factors that were associated with increasing pre‐diagnostic interval. We hypothesize that delay in diagnosis may be a reflection of underlying tumor biology. Rapidly growing tumors may result in significant clinical symptoms, especially in the setting of upper GI cancers, leading to early diagnosis, but often with advanced disease. Longer pre‐diagnostic interval may be a reflection of more indolent disease, which has been demonstrated in other tumor types [8, 19]. Patients seen during the COVID‐19 era appeared to be significantly older; however, their performance status and stage appeared to be similar. Furthermore, it is interesting that patients seen during the COVID‐19 pandemic had predominantly GEJ/esophageal primary rather than gastric. This may be due to the classic obstructive symptoms of cancers arising from esophageal and GEJ location prompting more urgent medical attention, whereas gastric cancer has a more insidious presentation [20, 21].

The COVID‐19 pandemic necessitated rapid pivoting of key resources toward patients with COVID‐19 while diverting from routine delivery of cancer care [22]. COVID‐19 infection leads to increased perioperative as well as long‐term mortality among patients with cancer [23]. Patients with gastroesophageal cancers frequently present at advanced stages and have high perioperative mortality as well as worse survival compared to other cancer types [24]. Recent large retrospective analysis of the National Cancer Database has shown that COVID19 contributed to underdiagnosis, resulting in stage migration among high‐risk gastrointestinal cancers, including esophageal and gastric [25]. Interestingly, similar to our data, the authors found no change in 1‐year survival or operative mortality. In contrast, retrospective data in Europe from the National Health Service and the Netherlands showed similar increased stage migration that also contributed to worse overall survival [16, 17]. These jurisdictional differences need to be further studied to understand if variations in pandemic response contributed to survival outcomes. This information will be critical to inform future pandemic responses. It is reassuring that for the majority of patients, our findings support an adequately functioning healthcare system, despite unprecedented challenges, that responded to patients seeking care during a worldwide pandemic. Specifically, metrics of health‐system delay, such as time to surgical resection, as well as time to PET/CT scan, were not numerically nor statistically different during the COVID‐19 pandemic compared to pre‐COVID. Although reassuring, newer models of cancer screening will be paramount to detect patients with more aggressive biology in order to diagnose them earlier in their disease trajectory and potentially improve outcomes.

## 5 Limitations

This study has several limitations. Foremost, this is a single‐center retrospective analysis and may not reflect cancer care delivery models and patient outcomes at other centres. Our cohort design could not account for non‐COVID‐19 pandemic‐related events that occurred during the study period. Symptom onset recall during the COVID‐19 pandemic may be exaggerated, and precise dates are limited by recall bias. Identifying cancer‐related symptom onset using electronic health records is challenging and is a reflection of the treating physician's interpretation. Even though our approach has clear drawbacks, this information is often not collected as part of larger registries, and we believe it is more accurate than using retrospective questionnaires. To minimize misattribution, two independent reviewers surveyed the charts and a standardized approach was used to approximate duration for cases with uncertain dates as well as the type of symptoms. Finally, we did not capture the full extent of health‐system impacts such as COVID positivity, perioperative morbidity, surveillance follow‐up/imaging, allied health involvement, supportive care delays, and use of virtual models of care. These will need to be studied as long‐term follow‐up data are accrued.

## 6 Conclusion

In conclusion, our study showed that resectable esophageal and gastric cancer patients diagnosed during the COVID19 pandemic had a statistically significant increase in their pre‐diagnostic interval. However, this increase did not contribute to worse overall survival in a multivariable analysis. Furthermore, we found no evidence of pandemic‐related health system delays in treatment, once a diagnosis was made, highlighting the importance of risk mitigation strategies during a global pandemic.

## Author Contributions

**Xin Wang:** conceptualization (lead), data curation (equal), formal analysis (equal), investigation (equal), methodology (equal), visualization (equal), writing – original draft (lead), writing – review and editing (lead). **Yvonne Bach:** data curation (equal), formal analysis (equal), methodology (equal), project administration (equal), writing – original draft (equal), writing – review and editing (equal). **Katherine Lajkosz:** data curation (equal), writing – review and editing (equal). **Osvaldo Espin‐Garcia:** formal analysis (equal), methodology (equal), visualization (equal), writing – review and editing (equal). **Hiroko Aoyama:** data curation (equal), investigation (equal), methodology (equal), writing – review and editing (equal). **Michael Wang:** data curation (equal), investigation (equal), writing – review and editing (equal). **Ronan McLaughlin:** data curation (equal), investigation (equal), writing – review and editing (equal). **Lucy Ma:** data curation (equal), investigation (equal), writing – review and editing (equal). **Carly Barron:** data curation (equal), investigation (equal), writing – review and editing (equal). **Farooq Abdul Rehman:** data curation (equal), investigation (equal), writing – review and editing (equal). **Eric Xueyu Chen:** data curation (equal), investigation (equal), writing – review and editing (equal). **Johnathan Chi‐Wai Yeung:** data curation (equal), investigation (equal), writing – review and editing (equal). **Carol J. Swallow:** data curation (equal), investigation (equal), writing – review and editing (equal). **Savtaj Brar:** data curation (equal), investigation (equal), writing – review and editing (equal). **Rebecca Wong:** data curation (equal), investigation (equal), writing – review and editing (equal). **Aruz Mesci:** data curation (equal), investigation (equal), writing – review and editing (equal). **John Kim:** data curation (equal), investigation (equal), writing – review and editing (equal). **Patrick Veit‐Haibach:** data curation (equal), investigation (equal), writing – review and editing (equal). **Sangeetha Kalimuthu:** data curation (equal), investigation (equal), writing – review and editing (equal). **Raymond Woo‐Jun Jang:** data curation (equal), investigation (equal), writing – review and editing (equal). **Elena Elimova:** investigation (equal), methodology (equal), project administration (equal), resources (equal), supervision (equal), writing – original draft (equal), writing – review and editing (equal).

## Disclosure

E.E. is a consultant for BMS, Zymeworks, Adaptimmune, Beigene, Jazz, Astellas, Virecta Tx, Signatera, Abbvie, Daiichi‐Sankyo, E.E. has received grant/research support from BMS, Zymeworks, Adaptimmune, Astra Zeneca, Jazz, Amgen. None relevant to this work. All other authors have no relevant disclosures.

## Conflicts of Interest

The authors declare no conflicts of interest.

## Data Availability

The data that support the findings of this study are available on request from the corresponding author. The data are not publicly available due to privacy or ethical restrictions.
